# Roles of Molecular Layer Interneurons in Sensory Information Processing in Mouse Cerebellar Cortex Crus II In Vivo

**DOI:** 10.1371/journal.pone.0037031

**Published:** 2012-05-18

**Authors:** Chun-Ping Chu, Yan-Hua Bing, Heng Liu, De-Lai Qiu

**Affiliations:** 1 Cellular Function Research Center, Yanbian University, Yanji, Jilin Province, China; 2 Department of Physiology and Pathophysiology, College of Medicine, Yanbian University, Yanji, Jilin Province, China; 3 Function Experiment Center, College of Basic Medicine, Yanbian University, Yanji, Jilin Province, China; Instituto de Neurociencias de Alicante UMH-CSIC, Spain

## Abstract

**Background:**

Cerebellar cortical molecular layer interneurons (MLIs) play essential roles in sensory information processing by the cerebellar cortex. However, recent experimental and modeling results are questioning traditional roles for molecular layer inhibition in the cerebellum.

**Methods and Main Results:**

Synaptic responses of MLIs and Purkinje cells (PCs), evoked by air-puff stimulation of the ipsilateral whisker pad were recorded from cerebellar cortex Crus II in urethane-anesthetized ICR mice by *in vivo* whole-cell patch-clamp recording techniques. Under current-clamp (I = 0), air-puff stimuli were found to primarily produce inhibition in PCs. In MLIs, this stimulus evoked spike firing regardless of whether they made basket-type synaptic connections or not. However, MLIs not making basket-type synaptic connections had higher rates of background activity and also generated spontaneous spike-lets. Under voltage-clamp conditions, excitatory postsynaptic currents (EPSCs) were recorded in MLIs, although the predominant response of recorded PCs was an inhibitory postsynaptic potential (IPSP). The latencies of EPSCs were similar for all MLIs, but the time course and amplitude of EPSCs varied with depth in the molecular layer. The highest amplitude, shortest duration EPSCs were recorded from MLIs deep in the molecular layer, which also made basket-type synaptic connections. Comparing MLI to PC responses, time to peak of PC IPSP was significantly slower than MLI recorded EPSCs. Blocking GABA_A_ receptors uncovered larger EPSCs in PCs whose time to peak, half-width and 10–90% rising time were also significantly slower than in MLIs. Biocytin labeling indicated that the MLIs (but not PCs) are dye-coupled.

**Conclusions:**

These findings indicate that tactile face stimulation evokes rapid excitation in MLIs and inhibition occurring at later latencies in PCs in mouse cerebellar cortex Crus II. These results support previous suggestions that the lack of parallel fiber driven PC activity is due to the effect of MLI inhibition.

## Introduction

Purkinje cells (PCs) are the most investigated neurons in the mammalian cerebellum. Their unique discharge of two distinct types of spike firing patterns, simple spikes and complex spikes, enables their positive identification under *in vivo* recording conditions. An assumption is that information coming from mossy fibers produces beam-like excitation of parallel fibers, which sequentially induces the activity of their innervated PCs [1], . However, more natural stimulation of afferent cerebellar cortical activation failed to produce “beam-like” excitation of PCs [3]–[7]. Instead, the peripheral stimuli induced patch-like patterns of excitation or inhibition in PCs [3], [4], [8] and the excitation patterns were found to be above the region of the activated granule cell layer [4], [7], [9], [10]. Under the stimulation conditions used in these experiments, all of the recorded PC responses were inhibitory. However, excitatory synaptic responses were uncovered when inhibition was blocked, a result consistent with recent modeling and experimental results suggesting that the inability of parallel fibers to directly drive PC firing may be due to the presence of feed-forward molecular layer inhibition [11]–[15]. Our results therefore support the proposal that the molecular layer inhibitory interneurons play a complex, subtle and perhaps more central role in PC responses to afferent input and therefore in the physiological and functional organization of the cerebellar cortex.

The molecular layer interneurons (MLIs) of the cerebellar cortex have historically been divided into basket and stellate cells [1], receiving excitatory input from parallel fibers and inhibitory input from other interneurons, and exerting GABAergic inhibition on PCs [16]–[20]. Basket cells are usually found in the inner third of the molecular layer and their somas are close or within PC layer, they are characterized by the basket-like structures that their axonal arborizations envelop PCs soma [1], [16], [21]–[23]. The stellate cells are usually located in the outer two thirds of molecular layer, and the inhibitory inputs of stellate cells directly innervate the dendrites of PCs [1], [16], [24]. Although basket and stellate cells have been further classified into several subtypes according to their exact axonal shape [1], [16], the separation of MLIs into two different classes has been challenged by recent studies [14], [25]–[28]. However, the stellate-type dendritic and basket-type somatic inhibition are predicted to play different functional roles and have different post-synaptic effects on PCs [14]. The stellate-type dendritic inhibition is predicted to specifically counterbalance the parallel fiber excitation in local regions of the PC dendrites [29]–[30], resulting in no direct influence on PC spiking output [13]. In contrast, basket-type somatic inhibition is powerful and rapid [22], [31], and results in direct influence on PC spiking output by inhibition of the soma and initial segment of PCs [13], [14], [22], [23], [31]. Although the model-based studies suggest that both stellate-type dendritic and basket-type somatic inhibition are involved in controlling PC responses to parallel fiber input [13], [14], the physiological roles and difference of stellate-type and basket-type MLIs in sensory information processing in mouse cerebellar cortex Crus II *in vivo* are not well understood.

Here, we used *in vivo* whole-cell patch-clamp recording with biocytin histochemistry to investigate the synaptic responses of cerebellar PCs and MLIs in response to tactile stimulation in urethane-anesthetized mice. Our results showed that air-puff stimuli were found to primary produce inhibition in PCs, but evoked spike firing regardless of whether they made basket connections or not in MLIs, in cerebellar cortex Crus II. Our results support previous suggestions that the lack of parallel fiber driven PC activity is due to the effect of MLI inhibition, and that structure of molecular layer circuitry supports a precise timing relationship and interaction between MLIs and PCs that is likely important for sensory information processing.

## Results

### Air-puff Stimulation of the Ipsilateral Whisker Pad Evoked Excitation in MLIs

**Figure 1:**
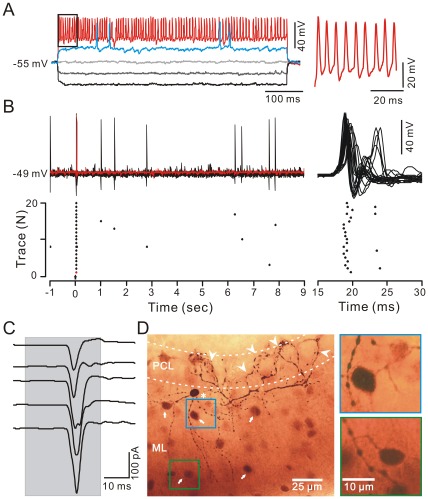
Air-puff stimulation of the ipsilateral whisker pad evoked reliable spike firing in basket-type MLIs. A, Left, whole-cell patch-clamp recording from a basket-type MLI in response to hyperpolarizing (−100 µA), followed by a series of depolarizing (+50 pA/step) current pulses. Right, enlarged trace from the quadrangle shown in the left panel. B, Left, under current-clamp (I = 0), superposition of 20 sequential traces (upper) and raster plot of spike firing (lower) showing the basket-type MLI in response to the air-puff stimulus (arrow, 30 ms). Right, enlarged traces (upper) and raster plot (lower) of left panel. Time point (0) denotes the onset of stimulus. C, Under voltage-clamp (V_hold_ = −70 mV) conditions, five consecutive traces demonstrate the air-puff stimulation (bar, 30 ms)-evoked EPSCs (right) in the basket cell. D, Left, a photomicrograph depicting the morphology of the basket-type MLI (asterisk) filled with biocytin. Note that the basket cell drops descending collaterals that wrap around at least five PCs soma (arrowheads) and is dye-coupled with a group of other MLIs (arrows). Right, magnified photomicrographs from the quadrangles in the left panel showing the dye-coupled stellate cells at different focal planes. PCL, Purkinje cell layer; ML, molecular layer.

**Figure 2:**
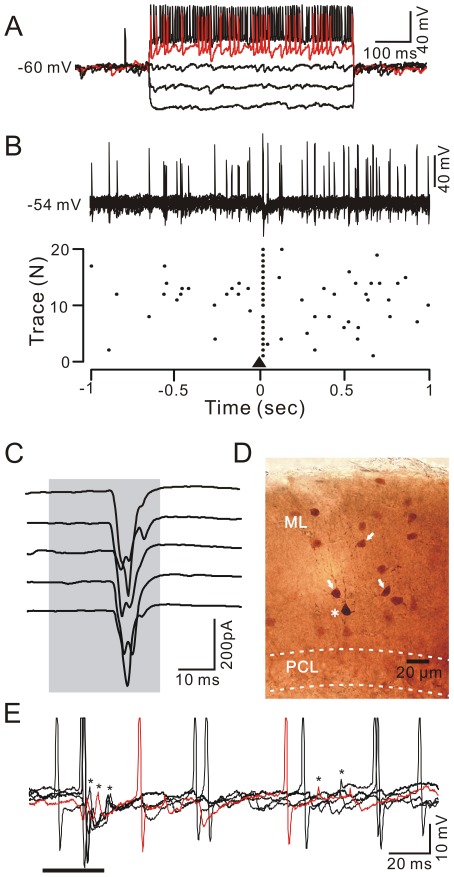
Air-puff stimulation of the ipsilateral whisker pad evoked spike firing in stellate-type MLIs. A, Whole-cell patch-clamp recording from a stellate-type MLI in response to hyperpolarizing pulses (−100 pA) followed by a series of depolarizing current pulses (+50 pA/step). B, Under current clamp (I = 0), superposition of 20 consecutive traces (upper) and a raster plot of spike firing (lower) showing the stellate-type MLI response to air-puff stimulation (black triangle, 30 ms). Time point 0 denotes the onset of the air-puff stimulation. C, Under voltage-clamp (V_hold_ = −70 mV), five sequential traces showing air-puff stimulation (bar, 30 ms)-evoked EPSCs in the stellate-type MLI. D, A photomicrograph depicting the morphology of the stellate-type MLI (asterisk) filled with biocytin and dye-coupled to several other MLI s (arrows). E, Under current-clamp conditions (n = 0), example traces (n = 5) showing the air-puff stimulation-evoked spike firing or spikelet discharge in a stellate-type MLI. Spikelet discharges are indicated by asterisks.

In total, 36 MLIs were recorded under whole-cell patch-clamp recording conditions and were identified by biocytin labeling. These MLIs possessed somas of 10.26±0.23 µm (*n* = 36 cells) in diameter. Under current-clamp (I = 0), they exhibited a low average rate of irregular spontaneous simple spiking activity (2.76±0.48 Hz; *n* = 36) at the resting potential (−54.7±0.6 mV; *n* = 36; Fig. 1B, 2B), but expressed high frequency spike firing (134.6±16.1 Hz; *n* = 36) in response to a depolarizing current pulse (100 pA; Fig. 1A, 2A). Spontaneous spikelet activity (Fig. 2E) was also observed in some of the MLIs at resting potentials, consistent with a previous study [26]. The mean value of the input resistance (R_input_) was 255.8±23.5 MΩ (*n* = 36), which was significantly higher than that for PCs (86.8±13.4 MΩ, *n* = 17; *P* = 0.00001). Notably, air-puff stimulation of the ipsilateral whisker pad evoked simple spike firing in 29 of 36 MLIs (Fig. 1B, 2B). The tactile stimulation-evoked excitation in the MLIs was completely blocked by 50 µM AMPA (α-amino-3-hydroxy-5-methyl-4-isoxazole propionate) receptor antagonist, NBQX, and abolished by 10 µM tetrodotoxin (TTX; not shown). Biocytin histochemistry revealed that the tactile stimulation-sensitive MLIs included basket-type (n = 11) and stellate-type (n = 25) MLIs, which were dye coupled to other MLIs with some distance of their somas (Fig. 1D, 2D).

### Distinct Properties of Basket-type and Stellate-type MLIs in Response to the air-puff Stimulation of Ipsilateral Whisker Pad

**Figure 3:**
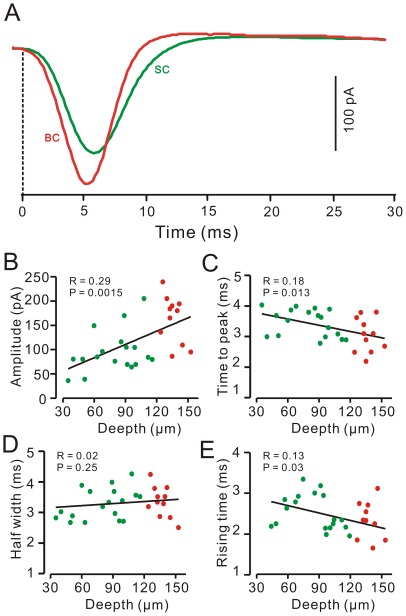
Relationships between properties of the evoked-EPSCs in MLIs and the depth of their somas location. A, Representative currents traces show the air-puff stimulation-evoked EPSCs in a basket-type MLI (BC; red) and a stellate-type MLI (SC; green). The time point (0) indicates the onset of the responses. B-E, Plots show the amplitude (B), time to peak (C), half-width (D) and 10–90% rising time (E) of EPSCs versus the depth of the somas in the molecular layer, respectively. The stellate-type MLIs are indicated by green color dots, and the basket-type MLIs are indicated by red color dots. The solid lines indicate linear regression (R).

The basket-type MLIs are found in the bottom 1/3 of molecular layer just above PCs, their soma diameter is 12.30±0.40 µm (*n* = 11 cells). Their identification depends on the presence of characteristic terminals that dropped descending collaterals to wrap around several somas and axon initial segments of PCs (Fig. 1D). In contrast, stellate-type MLIs possessed somas with a mean diameter of 9.13±0.16 µm (n = 25), which was significant smaller than basket-type MLIs (12.30±0.40 µm; *n* = 11; *P* = 0.01). However, the mean value of the R_input_ was 260.3.8±23.1 MΩ (*n* = 25), which was similar to the basket cells (245.1±24.2 MΩ; *n* = 11; *P* = 0.65). The stellate-type MLIs exhibited irregular spontaneous spike firing at a mean frequency of 4.26±0.78 Hz (Fig. 2B; *n* = 25), which was significantly higher than basket-type MLIs (0.08±0.02 Hz; *n* = 11; *p* = 0.005). Air-puff stimulation of the ipsilateral whisker pad evoked reliable spike firing in 11 of 11 basket-type MLIs (Fig. 1B) but in 18 of 25 stellate-type MLIs (Fig. 2B). Seven of 25 stellate-type synaptic connection MLIs exhibited an increase in spike firing rate and spikelet discharge in response to the stimulus: the mean frequency of spike firing within 50 ms after onset of responses was 13.60±0.92 Hz (*n* = 7), which was significantly higher than the baseline (4.69±0.91 Hz; *n* = 7; *P* = 0.0004; Fig. 2E). The mean amplitude of the evoked spikelet was 4.27±0.53 mV (*n* = 7). Under voltage-clamp conditions (V_hold_ = −70 mV), the stimulus evoked fast EPSCs (Fig. 1C) with a mean amplitude of 163.7±14.8 pA (*n* = 11) in basket-type MLIs, which was significant larger than the mean amplitude of EPSCs (96.42±14.3 pA; *n* = 18) evoked in stellate-type MLIs (Fig. 2C). However, there was no significant different in time to peak (BC: 3.04±0.25 ms; n = 11; SC: 3.45±0.46 ms; *n* = 18; *P* = 0.13) and half-width (BC: 3.43±0.42 ms; n = 11; SC: 3.17±0.30; n = 18; *p* = 0.42) of the evoked-EPSCs between basket-type and stellate-type MLIs. Further, we investigated the relationship between the properties of the evoked-EPSCs and the depth of MLIs somas in molecular layer using linear regression analysis. As shown in Figure 3, the amplitude of the EPSCs correlated positively with the depth of MLIs somas in molecular layer (Fig. 3B; *R* = 0.29; *P* = 0.0015), exhibited an increase with depth of MLIs somas in molecular layer. The time to peak of the EPSCs correlated negatively with the depth of MLIs somas in molecular layer (Fig. 3C; *R* = 0.18; *P* = 0.013), expressed a decrease with depth of MLIs somas in molecular layer. The half-width of the EPSCs expressed no significant correlation with the depth of MLIs somas in molecular layer (Fig. 3D; *R* = 0.0.02; *P* = 0.23). The 10–90% rising time of the EPSCs correlated negatively with the depth of MLIs somas in molecular layer (Fig. 3E; *R* = 0.13; *P* = 0.03), expressed a decrease with depth of MLIs somas in molecular layer. Taken together, the latencies of the evoked-EPSCs were similar for all MLIs, but the time course and amplitude of the EPSCs were varied with depth in the molecular layer. The highest amplitude, fastest EPSCs were recorded from MLIs deep in the molecular layer, which also made basket-type synaptic connections. These results consistent with modeling prediction, suggested differences in the dendritic tree of those basket-type connections, may reflect increased drive from ascending granule cell axons on these cells [14].

### MLIs Expressed More Rapid Responses than PCs

**Figure 4:**
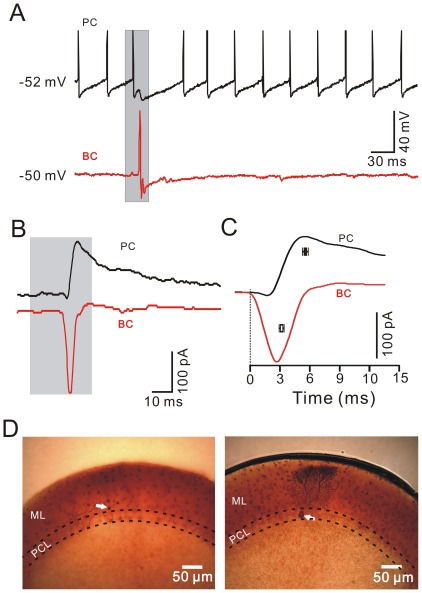
Comparison of the air-puff stimulation-evoked responses in a basket -type MLI **and a PC in the same mouse cerebellar Crus II.** A, Under current-clamp (I = 0) conditions, air-puff stimulation (grey shadow) evoked spike firing in a basket-type MLI (lower), and an IPSP with a pause in spike firing in a PC (upper), in the same mouse cerebellar Crus II. B, Under voltage-clamp (V_hold_ = −70 mV), air-puff stimulation (grey shadow) evoked fast EPSCs in the basket-type MLI (lower) and IPSCs in the PC (upper). C, Enlarged current traces from (B) and the mean values (± SEM) of the time to peak for the current traces evoked by air-puff stimulation in the PC (black; *n* = 5) and the basket-type MLI (red; *n* = 5). D, Consecutive photomicrographs showing the basket-type MLI (white arrow; left) and the PC (white arrow; right) filled with biocytin. The two recorded cells were apart from ∼150 µm in coronal plane. PCL, PC layer; ML, molecular layer.

**Figure 5:**
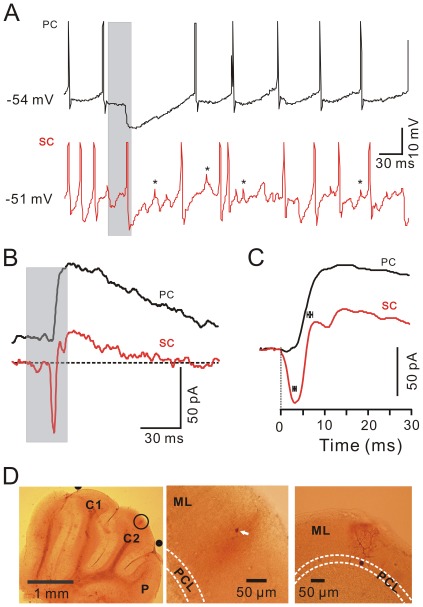
Comparison of the air-puff stimulation-evoked responses in stellate-type MLIs and PCs in the same mouse cerebellar Crus II. A, Under current-clamp (I = 0) conditions, the air-puff stimulation (grey shadow) evoked spike firing in a stellate-type MLI (lower), and an IPSP with a pause of spike firing in a PC (upper), in the same mouse cerebellar Crus II. Asterisks indicate spikelets discharge. B, Under voltage-clamp (V_hold_ = −70 mV), tactile stimulation (grey shadow) evoked fast EPSCs in the stellate-type MLI (lower) and IPSCs in the PC (upper). C, Enlarged current traces from (B) and the mean values (± SEM) of the time to peak for the current traces evoked by the air-puff stimulation in the PCs (black; *n* = 5) and the basket-type MLIs (red; *n* = 5). D, Photomicrographs show the morphology of the cells in A-C. The left column shows an overview of the location of the biocytin-labeled stellate-type MLI, which is indicated with a black circle in the left photomicrograph. The middle column shows the detail of the biocytin-labeled stellate-type MLI. The right column shows the detail of the biocytin-labeled PC. The two recorded cells were apart from ∼300 µm in coronal plane. PCL, PC layer; ML, molecular layer.

The time courses of the evoked responses in MLIs and PCs were compared by arbitrarily recording them (100–500 µm in distance) in the same cerebellar cortex Crus II (Fig. 4D, 5D). The stimulation evoked spike firing in MLIs and IPSP in the PCs (Fig. 4A, 5A). The latency of the responses was 16.31±0.22 ms (*n* = 10) for MLIs, which was no significant different from the latency for PCs (16.37±0.23 ms; *n* = 10; *P* = 0.86; paired Student’s t test). However, the evoked spike in the basket-type MLIs peaked at 19.72±0.68 ms (*n* = 5) after onset of stimulation, which was significantly earlier than the onset of evoked IPSP in PCs (20.64±0.59 ms; *n* = 5; *P* = 0.0095; paired Student’s t test). The difference between the evoked spike firing in basket-type MLIs and the onset of evoked IPSP in PCs was 0.92±0.17 ms (*n* = 5), suggested that tactile stimulation-evoked spike firing in basket-type MLIs resulted in a perisomatic inhibition of PCs within ∼1 ms. In contrast, the evoked spike in the stellate-like MLIs peaked at 20.62±0.69 ms (*n* = 5) after onset of stimulation, which was not significantly different from the onset of the evoked IPSP in PCs (20.72±0.49 ms; *n* = 5; *P* = 0.68; paired Student’s t test). Under voltage-clamp conditions (V_hold_ = −70 mV), the air-puff stimulus evoked fast EPSCs in the MLIs but induced a tiny inward currents followed by strong IPSCs in the PCs (Fig. 4B, 5B). The strong IPSCs expressed outward currents which were not reversed at the holding potential of –70mV, suggesting poor space clamping of PCs under *in vivo* conditions. The cerebellar PCs express large soma, abundant dendrites and very long axons, especially in the intact cerebellum, therefore, the somatic membrane potential may be well controlled, but the membrane potential of the axon initial segment may be poorly controlled [13]. The time-to-peak of the evoked EPSCs was 3.26±0.37 ms (*n* = 10) in the MLIs, which was significantly faster than that in the PCs (5.79±0.50 ms; *n* = 10; *P* = 0.004; Fig. 4C, 5C).

**Figure 6:**
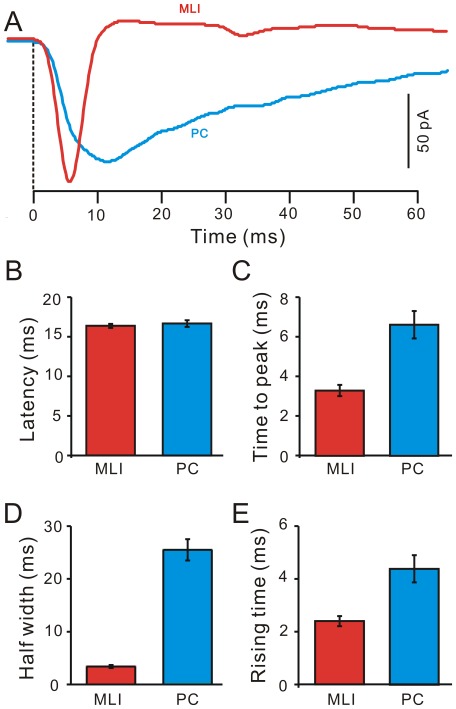
Properties of the air-puff stimulation-evoked EPSCs in MLIs and PCs. A, Representative currents traces showing the air-puff stimulation-evoked EPSCs in a MLI (MLI; red) and a PC (blue). The EPSCs in the PC were evoked in the presence of SR95531 (20 µM), a GABA_A_ selective antagonist. The time point (0) indicates the onset of the responses. B, Bar graph showing the mean amplitude of the EPSCs evoked by the air-puff stimulation in the MLIs (MLI; red; n = 10) and the PC (blue; n = 10). C, Summary of data showing the time to peak for the EPSCs in the MLIs (MLI; red; n = 10) and the PC (blue; n = 10). D, Pooled data showing the half-width of the EPSCs in the MLIs (MLI; red; n = 10) and the PC (blue; n = 10). E, Summary of data showing the 10–90% rising time of the EPSCs in the MLIs (MLI; red; n = 10) and the PC (blue; n = 10).

Moreover, we compared the properties of the evoked EPSCs in PCs and in MLIs. As shown in Figure 6A, the stimulation-evoked EPSCs in PCs were obtained in the presence of the GABA_A_ receptor antagonist, SR95531 (20 µM). The latency of the EPSCs in PCs was 16.68±0.42 ms (*n* = 8), which was not significantly different from that in the MLIs (16.36±0.35 ms, *n* = 29; *P* = 0.63; Fig. 6B). However, the time to peak of the EPSCs in PCs was 6.63±0.70 ms (*n* = 8), which was significantly slower than that in the MLIs (3.30±0.25 ms; *n* = 29; *P* = 0.002; Fig. 6C). The half-width of the EPSCs in PCs was 25.46±2.01 ms (n = 8), which was significantly wider than that in the MLIs (3.28±0.30 ms; *n* = 29; *P*<0.00001; Fig. 6D). The 10–90% rising time of EPSCs in PCs was 4.39±0.52 ms (n = 8), which was significantly slower than that in the MLIs (2.40±0.18; *n* = 29; *P* = 0.003; Fig. 6E). These data indicated that blocking GABAergic inhibition revealed large EPSCs in PCs not as readily apparent when inhibition was intact. However, the EPSCs evoked in PCs expressed much more slowly than in the EPSCs evoked in MLIs. The blockade of GABAergic inhibition is maybe included the granule cell layer inhibition, which may also contribute to the large EPSCs evoked by the tactile stimulation.

## Discussion

In the present study, we found that air-puff stimulation of ipsilateral whisker pad evokes responses in both MLIs and PCs in mouse cerebellar cortex Crus II. MLIs responded with rapid excitation, whereas PC responses were dominated by inhibition occurring at later latencies. The highest amplitude, fastest EPSCs were recorded from MLIs deep in the molecular layer, which also made basket-type synaptic connections.

MLIs of cerebellar cortex were originally described as two separate molecular layer cell classes, so-called basket and stellate cells, which were distinguished by their specific inhibitory axon collaterals [1]. Basket cells are usually found in the inner third of the molecular layer and their somas are close or within PC layer [1], they are characterized by the basket-like structures that their axonal arborizations envelop PCs soma to form pinceau synapses and provide a strong basket-type somatic inhibition of the PCs [1], . Stellate cells do not display axosomatic contacts and are usually found in the middle and outer part of the molecular layer and their inhibitory input directly to the dendrites of PCs [1], [16], [24]. Consistent with previous reports [1], [16], [21]–[23], our biocytin histochemistry analysis indicated that the basket cells were located in the inner of molecular layer and their somas are close to PCs, their basket-like axonal arborizations enveloped PCs soma. However, a clear separation of MLIs into two distinct classes has been challenged by recent morphological and physiological studies [14], [25]–[28]. Using principal component analysis, Sultan and Bower [25] have shown that basket and stellate cells varied continuously in their morphology depending on the location of the soma in the molecular layer. Anatomical and modeling studies of MLIs have suggested that basket and stellate cells are one homogenous population of cells, a MLI makes a basket-type connection was simply related to the depth of the soma in the molecular layer [14], [25]. Here, we used *in vivo* whole-cell recording with biocytin staining technique to study the spontaneous activity of MLIs. Our results showed that all the MLIs share properties of the resting potential, half-width of spikes, input resistance and dye-coupling to other MLIs in common. Although the stellate-type MLIs had higher rates of background activity, all the MLIs exhibited irregular spontaneous spike firing at resting potential and expressed similar high frequency firing in response to depolarizing current pulses. Importantly, all the MLIs expressed rapid excitation in response to the air-puff stimulation of ipsilateral whisker pad. Linear regression analysis of the relationship between the properties of the evoked-EPSCs and the depth of MLIs somas in molecular layer revealed that the time course and amplitude of the evoked-EPSCs were varied with depth in the molecular layer. The highest amplitude, fastest EPSCs were recorded from MLIs deep in the molecular layer, which also made basket-type synaptic connections. These results consist with previous studies [14], [25]–[28], suggesting that both stellate-type and basket-type MLIs are one homogenous population of cells, whose response properties change depending on the position of its soma in the molecular layer.

The functional organizational studies indicate that the MLIs are activated by the information coming from the mossy fiber-granule cell-parallel fiber pathway [16]. Under *in vivo* conditions, activity of vestibular primary afferent mossy fibers during ipsilateral roll tilt can increase the spike firing of basket and stellate cells [38]. Our results showed that air-puff stimuli on ipsilateral whisker pad evoked spike firing in MLIs, and the evoked responses were blocked by AMPA receptor antagonist, NBQX, indicated that the tactile stimulation information excited MLIs via the mossy fiber-granule cell-parallel fiber pathway [16], [38]. The MLIs receive the excitatory inputs from granule cell axons that produce two types of inhibition have distinctly different postsynaptic effect on PCs [14], [16], [32], [25]. The powerful and rapid basket-type somatic inhibition is predicted that to result in direct influence on PC spiking output by inhibition of their somas and initial segments [13], [14], [22], [31]. However, the weak stellate-type dendritic inhibition is predicted to counterbalance the parallel fiber excitation in local regions of the PC dendrites and to result in no direct influence on PC spiking output [13], [14], [29], [30], [33].

In the current as well as previous studies [12] air-puff stimulation of the ipsilateral whisker pad failed to provoke strong EPSCs and spike firing in PCs. Instead, the response of PCs was dominated by rapid activation of strong GABAA receptor-mediated inhibitory postsynaptic currents. Notably, the MLIs responded with rapid excitation, the highest amplitude, fastest EPSCs evoked by air-puff stimulation were basket-type MLIs. These results suggest that the IPSCs recorded in the PCs are due to activity in the MLI's and in particular, in those basket-type MLIs. Our present results were supported by several anatomical and physiological evidences. First, the MLIs are small, have high input resistance, exhibit a low threshold for activation ([24], [34], this study) and can be reliably triggered to spike with a sub-millisecond delay by a single parallel fiber input [35], [36], the spike firing of molecular layer interneurons could be activated by stimulation of a single granule cell, and was strongly influenced by individual quanta release from parallel fibers [36]. In contrast, PCs are large, have low input resistance. It is estimated that 50 simultaneously active granule cells are need to excite a PC [35]. Second, the MLIs are electrically coupled: an input to one interneuron can activate a group of interneurons through gap junctions [26]. Third, the basket-type inhibition on PCs is rapid and profound [13], [14], [22], [31], and the MLIs whose somas are deep in the molecular tayer have receptive fields similar to those of nearby and not distant regions of the granule cell layer [28], [37], [38], these cells might preferentially receive inputs from the ascending branch of the granule cell axon [14]. Finally, our present data showed the time to peak of the evoked-spike in the basket-type MLIs was significantly earlier than the onset of evoked IPSP in PCs, indicated that the IPSP recorded in PCs were induced by basket-type MLIs, suggesting that the stimulation-evoked spike firing in basket-type MLIs and resulted in a rapid perisomatic inhibition of PCs [13], [14], [23], [31].

In addition, the inhibitory axon of Golgi cell terminals to cerebellar glomeruli, the principle sites of mossy fiber termination on granule cells, therefore, Golgi cells modulate the activity of thousands of granule cells [38] but may not contribute to the tactile stimulation-evoked inhibition of PC. The PC axon recurrent collaterals were unlikely to contribute to the tactile stimulation-evoked inhibition of PC. Because no PCs were excited by the tactile stimulation in mouse cerebellar Crus II ([11], [12] this study) and the IPSCs induced in the PCs are too fast to be a result of PC axon collaterals. Collectively, our results consistent with modeling studies [13], [14], [25], [29], [30], suggested that stellate-type dendritic and basket-type somatic inhibition play different functional roles. The tactile stimulation induced IPSP and pause of spike firing is due to the direct influence of the powerful and rapid basket-type inhibitory inputs on the somas and initial segments of the PCs. On the other hand, the stellate-type MLIs might contribute to shunting inhibition of PCs dendrites, preventing the parallel fiber excitatory inputs flowing into the somas of PCs, and counterbalancing the parallel fiber excitation in local regions of the PC dendrites.

Moreover, urethane was preferred to the mixture of ketamine/xylazine and barbiturates for anesthesia, because both of them strongly affect neuronal synaptic transmission, especially GABA_A_ receptor-mediated synaptic transmission [39]–[41]. However, urethane depresses neuron excitability, without effects on excitatory glutamate mediated or inhibitory synaptic transmission [42].

One of the ongoing controversies in the physiological organization of molecular layer circuitry is the role played by parallel fibers in the response properties of PCs [1], [2]. While it has generally been assumed that parallel fibers directly drive PC output, results with tactile stimulation of the sort used in this study have consistently failed to find the expected 'beams' of active PCs [3]–[7], and instead have reported much more restricted regions of excited PCs [3], [4], [8]. Consistent with this result, we have previously reported [11], [12] and report again here that, in the mouse cerebellum, the dominant form of PC responses to air-puff stimuli are inhibitory. In fact, in this and previous studies [11], [12] we have failed to find excitatory PC responses likely because we have not specifically identified the location of granule cell activation. In rats, it has been shown that the only PCs that respond with short latency excitatory responses are located over activated regions of the granule cell layer, and even some PCs over that activated region respond primarily with inhibition [4], [7], [9], [10]. Consistent with several other recent reports in rats [4], [7], [9], [10], [13], [14], the data presented here suggests that PCs do receive excitatory inputs, but that their response to those inputs is blocked by molecular layer inhibition. Accordingly, our results are consistent with the hypothesis that the lack of parallel fiber induced beams of PCs results from the activity of MLIs. Further, our comparison of the timing of activity in MLIs and PCs in nearby regions of Crus II demonstrate that the organization of molecular layer circuitry as well as the biophysical properties of MLIs result in a rapid afferent activation of MLIs followed by a rapid induction of inhibition in PCs. These findings are consistent with recent network modeling studies [13], [14], [25], [29], [30] and are also consistent with the model-based suggestion that basket type somatic inhibition performs a different functional role than stellate-type dendritic inhibition [13], [14].

Taken together, our data indicate that tactile face stimulation evokes responses in both MLIs and PCs in mouse cerebellar cortex Crus II. MLIs responded with rapid excitation, whereas PC responses were dominated by inhibition occurring at later latencies. These results suggested that the lack of parallel fiber driven PC activity is due to the effect of MLI inhibition, and that structure of molecular layer circuitry supports a precise timing relationship and interaction between MLIs and PCs that is likely important for sensory information processing.

## Materials and Methods

### Anesthesia and Surgical Procedures

The anesthesia and surgical procedures have been described previously [11], [12]. In brief, the experimental procedures were approved by the Animal Care and Use Committee of Jilin University and were in accordance with the animal welfare guidelines of the U.S. National Institutes of Health. The permit number is SYXK(Ji)2007-0011. Adult (6–8-week-old) HA/ICR mice were anesthetized with urethane (1.3 g/kg body weight i.p.). A watertight chamber was created and a 1–1.5 mm craniotomy was drilled to expose the cerebellar surface corresponding to Crus II. The brain surface was constantly superfused with oxygenated artificial cerebrospinal fluid (ACSF: 125 mM NaCl, 3 mM KCl, 1 mM MgSO_4_, 2 mM CaCl_2_, 1 mM NaH_2_PO_4_, 25 mM NaHCO_3_, and 10 mM D-glucose) with a peristaltic pump (Gilson Minipulse 3; Villiers, Le Bel, France) at 0.4 ml/min. Rectal temperature was monitored and maintained at 37.0±0.2°C using body temperature equipment.

### Stimulation and Drug Application

Tactile stimulation of the ipsilateral whisker pad was performed by air-puff (30 ms, 50 psi) through a 12-gauge stainless steel tube connected to a pressurized injection system (Picospritzer® III; Parker Hannifin Co., Pine Brook, NJ). Air-puff was delivered to the ipsilateral C2–C3 whisker pad at 0.33 Hz, with synchronized electrophysiology recording via a Master 8 controller (A.M.P.I., Jerusalem, Israel) and Clampex 8.1 software (Molecular Device, Foster City, CA). All drugs were dissolved in ACSF and applied onto the cerebellar surface at 0.4 ml/min. NBQX (2,3-dioxo-6-nitro-1,2,3,4- tetrahydrobenzo[f] quinoxaline-7- sulfonamide) and SR95531 hydrobromide (6-imino-3-(4-methoxyphenyl)-1 (6H)-pyridazinebutanoic acid hydrobromide) were purchased from Tocris Cookson (Bristol, UK). Tetrodotoxin was purchased from Sigma (Sigma-Aldrich, Shanghai, China).

### Whole-cell Recording and Biocytin Histochemistry

The whole-cell recording and biocytin histochemistry procedures have been described previously [12]. In brief, the *in vivo* whole-cell patch-clamp recordings from basket and stellate cells were performed using an Axopatch-1D amplifier (Molecular Device, Foster City, CA). Patch pipettes were made with a puller (PB-10; Narishige, Tokyo) from thick-wall borosilicate glass (GD-1.5; Narishige). They were filled a solution consisting of 120 mM potassium gluconate, 10 mM HEPES, 1 mM EGTA, 5 mM KCl, 3.5 mM MgCl2, 4 mM NaCl, 8 mM biocytin, 4 mM Na_2_ATP and 0.2 mM Na_2_GTP (pH adjusted to 7.3 with KOH). Patch-pipettes were mounted using a micromanipulator (MP-285, Sutter Instrument Company, Novato, CA). The patch pipette resistances were 6–7 MΩ in the bath, with series resistances in a range of 10–40 MΩ, compensated by 80%. The depth of the recorded cell was roughly obtained from the display value of the micromanipulator. The MLIs and PCs were roughly identified by spontaneous spike activity and the depth of the recording site before the whole-cell patch-clamp recording was performed. PCs were distinguished from MLIs by their location in the Purkinje cell layer, as well as the presence of climbing fiber type discharges [11], [12]. All recorded MLIs were biocytin filled, and therefore could be distinguished by the presence of their somas within the molecular layer and the characteristic shapes of their dendrites. The biocytin fills were also used to identify those MLIs making basket-type somatic connection. For comparing the time course of the responses evoked by air-puff stimulation in molecular layer interneurons and PCs, a molecular layer interneuron and a PC were recorded in the same cerebellar cortex Crus II with a distance of 100–500 µm. After electrophysiological recording, the whole brain was removed and fixed in 4% paraformaldehyde in 0.1 phosphate buffer (PB). Cerebellar slices were cut in the sagittal plane at 200 µm using a vibratome (XY-86, ZheJiang, China). Biocytin was detected using 3,3′-diaminobenzidine tetrahydrochloride histochemistry.

### Statistical Analysis

Input resistance (R_in_) was calculated from steady-state voltage deflections during 500 ms step hyperpolarizing current injection (50 pA). The electrophysiological data were analyzed using Clampfit 8.1 software. Values are expressed as the mean ± SEM. Differences between the mean values recorded under control and test conditions were evaluated with the Student’s paired t-test, ANOVA and linear regression analysis using SPSS (Chicago, IL) software.
